# A compelling need to empirically validate bipolar depression

**DOI:** 10.1186/s40345-023-00295-7

**Published:** 2023-04-28

**Authors:** Diego J. Martino, Marina P. Valerio

**Affiliations:** 1 National Council of Scientific and Technical Research (CONICET), Charcas 4189, 1º “C” (1425), Buenos Aires, Argentina; 2 Institute of Cognitive and Translational Neuroscience (INCyT), INECO Foundation, Favaloro University, Buenos Aires, Argentina; 3 Psychiatric Emergencies Hospital Torcuato de Alvear, Av. Warnes 2630, Buenos Aires, Argentina

Dear editor,

We read with great interest the paper by Ghaemi et al. (2022) entitled “Clinical research diagnostic criteria for bipolar illness (CRDC-BP): rationale and validity” recently published in the International Journal of Bipolar Disorders. Essentially the authors highlight the need for “new, purely research-based diagnostic criteria aimed primarily at validity” as “essential to the success of biological and pharmacological research”. Thus, they propose a series of diagnostic criteria marking a counterpoint to the Research Diagnostic Criteria in 1978, the immediate antecedent of DSM-III (1980), which focused on improving reliability rather than validity. We welcome that a group of prominent researchers emphasize the need for valid diagnoses based on empirical data as a primary condition for advancing our knowledge on bipolar disorder (BD). However, we would like to contribute with an additional perspective regarding the current clinical construct of bipolar depression.

The authors mention that the validity of major depressive disorder (MDD) has never been proven, and draw attention to the low reliability (kappa = 0.28) of this diagnosis in DSM-5 field trials (Freedman et al. 2013). In fact, there is currently some consensus that MDD does not delimit a single illness with a causal mechanism, pathophysiology, prognosis, or response to treatment (Sanacora 2020). On the contrary, it includes heterogeneous depressive experiences, from normal extreme reactions of intense sadness in the face of stressful life events to a highly recurrent and debilitating illness (Lorenzo-Luaces 2015). However, there is no logical argument for supposing that the same set of diagnostic criteria of major depressive episode (MDE) defines a more homogeneous clinical construct in the field of BD than MDD (Martino and Valerio 2021). In a series of recent small exploratory studies, we found that BD patients with melancholic and non-melancholic depressions differed on a number of external validators such as clinical features, neurocognitive performance, familial aggregation, clinical course, and psychosocial functioning (Martino and Valerio 2022; Martino et al. 2022; Valerio et al. 2022; Valerio et al. 2023). These preliminary data would suggest the need for more and better clinical research before concluding on the validity of the current concept of bipolar depression. On the other hand, although the reliability of type I BD (kappa = 0.58) was better than that of MDD in the DSM-5 field trials (for the accurate identification of manic episodes), it fell below acceptable values in the case of BD type II (kappa = 0.40) and, presumably, would fall more in diagnosis suggested by the authors of the review such as subthreshold hypomania (two days duration) or bipolar spectrum depressions (Freedman et al. 2013). Altogether, there are limited empirical data to support that MDE in the context of BD is any more valid or more reliable than that of MDD.

The authors also argue “the common criticism that broad definitions of bipolar illness are harmful ignores the equally valid criticism that broad definitions of MDD are harmful”. They point out that in the pre-DSM-III era recurrent depressions were part of manic-depressive illness (MDI) and that now some genetic studies have shown an overlap between DSM-defined MDD and BD, contradicting foundational research in the 1970 that separated both entities. So, they seem to propose that part of the MDD might be misdiagnosed and that, based on hypomania of shorter duration (e.g., for BD type II) or bipolarity specifiers/predictors (e.g., for mixed depression or bipolar spectrum depressions), they would be returned to the BD field (presumably in their opinion improving the validity of both MDD and BD). However, these statements deserve certain nuances. First, the current definition of bipolar depression is also broader than in the pre-DSM-III era in some aspects: it was classically conceptualized as melancholic/psychotic and recurrent, but after that expert consensus in the late 1970s any depressive episode (melancholic/psychotic or non-melancholic/nonpsychotic, whether recurrent or not) that occurred in a subject with a history of elevated mood episode was considered "bipolar" (Martino and Valerio 2021). This change was not based on any empirical data, and our recent preliminary findings do not support it (Martino and Valerio 2022; Martino et al. 2022; Valerio et al. 2022; Valerio et al. 2023). Thus, comparisons of studies involving the concept of bipolar depression before and after the publication of the DSM-III should be carried out with caution since they address different clinical pictures. Likewise, if MDD has not proven to be a valid diagnosis, “definitive” evidence of overlap with BD from genetic studies might depend on a mere artifact by not using phenotypes that carve nature at its joints. We do agree with Ghaemi and colleagues that the MDD diagnosis is too broad. Some cohort studies have shown that more than 40% of healthy subjects meet MDD criteria (i.e. MDE) at some point in a 15–25 year follow-up period (Moffit et al. 2010; Rohde et al. 2013). Thus, the lack of validity of MDD appears to far exceed some misdiagnosed cases of true BD, presumably including episodes that would be normal adaptive responses related to life conditions rather than real mental disorders (Lorenzo-Luaces 2015; Allen et al. 2014). Similarly, prospective studies in healthy subjects showed prevalences of subthreshold hypomania around 20–30% at the end of 10–20 years' follow-up, most of which occur in subjects who also suffered from major or minor depression in that period (Angts et al. 2003; Zimmermann et al. 2009). It could be hypothesized that while some of these common subthreshold features may help identify true BD (as the authors suggest), others may be transient homeostatic responses, or nonspecific markers of psychopathology of several clinical conditions highly comorbid with DSM-defined MDD (such as personality disorders, substance use disorders, ADHD, or conduct disorder) (Lewinsohn et al. 2000; Tijjsen et al. 2010). Even if this were the case, the results of some of the main studies cited by the authors using bipolarity specifiers (e.g. Angst et al. 2011) would be explained by the proper relocation of some true BD patients. The heterogeneity of the MDD could be one of the reasons why large studies using bipolarity specifiers/predictors have difficulties demonstrating their usefulness in predicting conversion to DSM-BD or poor response to antidepressants (Zimmermann et al. 2009; Perlis et al 2011). Therefore, the problem would not be the criteria for subthreshold hypomania or the specifiers/predictors of bipolarity but their application to an invalid and unreliable diagnosis such as MDD (or MDE), which could contribute to a broader but equally invalid, unreliable and harmful diagnosis of BD.

Returning to the need for "purely research-based diagnostic criteria aimed primarily at validity", Ghaemi et al. stand out: “If the clinical phenotype for bipolar illness is wrong, imprecise or heterogeneous, genetic studies will fail, biological marker studies will be inconsistent, and treatment studies will be ineffective”. Although we fully agree with this premise, in our opinion, the validation based on empirical data of the current concept of bipolar depression (i.e. MDE in BD derived with minimal changes of the Research Diagnostic Criteria) is a cornerstone of their compelling and relevant purpose. Omitting this step, even knowing the heterogeneity and imprecision of the current clinical construct of MDE, might undermine advancing our understanding of the pathophysiological bases and the therapeutic approach of the full spectrum of BD (from BD type I to soft bipolar presentations). So, let us include some additional questions to their non-exhaustive list of future possibilities: Are all forms of depressive experience (melancholic/psychotic or non-melancholic/non-psychotic, recurrent or non-recurrent) equally relevant to the diagnosis of BD, or can some of them be considered only comorbid phenomena with hypo/mania? Do they all have the same response to the usual treatments? These are questions that were neglected for more than four decades and should now be part of any research program aimed at improving the validity of the BD.

## Data Availability

Not applicable.
